# ORAL HEALTH: THE MOST UNMET HEALTH CARE NEED FOR MEDICALLY VULNERABLE ELDERLY WITH OR WITHOUT DEMENTIA

**DOI:** 10.1093/geroni/igad104.2424

**Published:** 2023-12-21

**Authors:** maryam Tabrizi, Wei-Chen Lee

**Affiliations:** University of Nevada in Las Vegas, Las Vegas, Nevada, United States; University of Texas Medical Branch, Galveston, Texas, United States

## Abstract

Individuals aged 65 and older are living longer with multiple chronic conditions, leading to a larger population of medically vulnerable elderly with or without dementia. Consequently, only an interdisciplinary health system can meet the comprehensive health needs for older adults. Historically, Oral Health (OH) is the most unmet need in healthcare, particularly in older adults. This is due to several barriers including Interprofessional Education (IPE) and Interprofessional Collaboration (IPC). Therefore, there is a growing gap between OH and overall health. We conducted two studies in the past 4 years. The first study included pre-doctorate healthcare students (Medical, Nursing, Dental and Pharmacy) evaluating their IPE during their educational training and the influence of their education later as practitioners. The result of this study revealed an unsatisfactory IPE that would lead to minimum or no interdisciplinary collaboration. The second study (2019 to 2022) included dental residents in a one year post graduate program “Advanced Education in General Dentistry” (AEGD). In each year we collected a survey at the beginning of the residency program asking the level of comfort treating medically vulnerable elderly as a provider in a health team. We then exposed them to a comprehensive geriatric oral health both didactics and clinical skills. The same survey was again conducted at the end of the residency. The study revealed adequate training increased the level of residents’ ability to provide care within a health team. Interdisciplinary health services require interdisciplinary educational training to close the gap between oral health and overall health.

